# Supplementary figures and images for: Fungal infection, decline and persistence in the only obligate troglodytic Neotropical salamander
Source: PeerJ. 2020 Sep 22;8:e9763. doi: 10.7717/peerj.9763 (PMC7518159; doi:10.7717/peerj.9763)

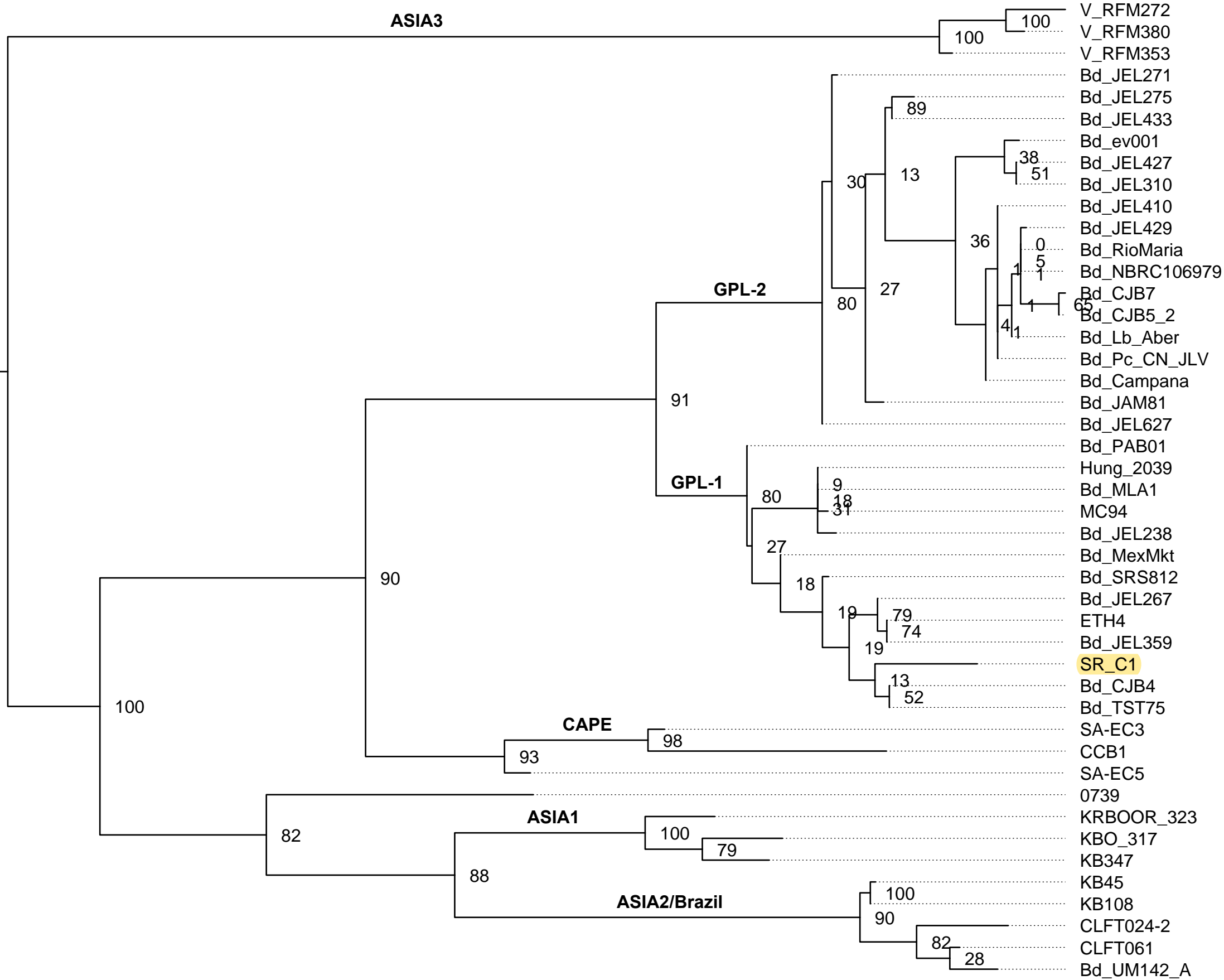

0 . 0 0 2

Supplement: Supplemental Information 1 — The sample from C. magnipes is within the Bd-Global Pandemic Lineage 1 (GPL-1) subclade. [file peerj-08-9763-s001.pdf]
